# P17 Quality assurance in bacterial identification and antimicrobial susceptibility testing results: an essential component of antimicrobial resistance surveillance

**DOI:** 10.1093/jacamr/dlac053.017

**Published:** 2022-05-31

**Authors:** Susan Githii, Jedida Kahura, Mercy Zawadi, George Muchiri, Ethel Waswa, Richard Stabler

**Affiliations:** 1 Ministry of Health, Kenya; 2 London School of Hygiene & Tropical Medicine

## Abstract

**Background:**

Antimicrobial resistance (AMR) is a major threat to human health. Bacterial identification and antibiotic susceptibility testing play an essential role in patient care and control of antibiotic resistance mechanisms by indicating which antibiotics are most likely to treat an infection, reducing the empirical prescription of broad-spectrum antibiotics and avoiding the unnecessary prescription of antibiotics to reduce healthcare costs. AMR surveillance in Kenya has historically been focused on pathogens that cause significant mortality and morbidity such as diarrhoea illnesses and bacteraemia. Many private hospitals and a few public hospitals in Kenya have the capacity to conduct antimicrobial susceptibility testing; however, the available data are rarely verified for quality.

**Methods:**

This study was conducted in five sentinel sites in Kenya. Clinical samples were processed in the sites as part of routine microbiology. Each sentinel site selected isolates as per the National AMR sample referral guide and sent them to the National Microbiology Reference Laboratory (NMRL) for quality assessment. Upon reception of isolates at NMRL, they were subcultured and subjected to organism identification testing using MALDI-TOF equipment. Antimicrobial susceptibility testing was performed using Vitek 2 compact. In case of any discrepancy on organism identification, correct antibiotic selection and resistance interpretation, the sites were informed.

**Results:**

Antimicrobial susceptibility testing was conducted on *Escherichia coli* and *Staphylococcus aureus*, which were the most frequently isolated pathogens. High resistance to cephalosporins was observed with the *E. coli* isolates. Over 80% susceptibility of *E. coli* to amikacin, ampicillin, gentamicin, meropenem, nitrofurantoin and piperacillin/tazobactam was observed. This shows that the antibiotics can be used for empirical therapy in areas where there is no laboratory capacity for antibiotic susceptibility testing. Resistance to gentamicin of *S. aureus* isolates was found to be 10%, which shows that gentamicin still finds a role in controlling *S. aureus* infections. Penicillin resistance in *S. aureus* was 100%. High levels of resistance were observed with erythromycin (61%) and ampicillin (84%)

**Conclusions:**

There is no site that scored 100% in pathogen identification and drug–bug combination. Continuous capacity building of AMR surveillance sites through mentorships to improve quality of data generated is of paramount importance. *E. coli* and *S. aureus* were the most frequently isolated pathogens. High levels of resistance were observed with erythromycin (61%) and ampicillin (84%), which calls for antibiotic stewardship programmes to educate on optimal antibiotic use.

